# Supplementary material for: Overexpression of the High-Affinity Nitrate Transporter OsNRT2.3b Driven by Different Promoters in Barley Improves Yield and Nutrient Uptake Balance
Source: Int J Mol Sci. 2020 Feb 15;21(4):1320. doi: 10.3390/ijms21041320 (PMC7072886; doi:10.3390/ijms21041320)
Supplement: Supplementary file 1 [file ijms-21-01320-s001.pdf]

Table S1. Primers used to amplify the *OsNRT2.3b* open reading frame and identification primers of hygromycin.

Gene name	Primer	Sequence
OsNRT2.3b	5' (F)	GAGATGGAGGCTAAGCCGGTGGCG
	5' (R)	TTCTTCACAC CCCGGCCGGC GACG
Hygromycin	5'(F)	CTTCTGCGGGCGATTTGT
	5''(R)	CAGCGTCTCCGACCTGAT

Table S2. Primers used for quantitative real-time polymerase chain reaction.

Gene name	Forward primer (5'to 3')	Reverse primer (5'to3')
HvActin	AGCAACTGGGATGACATGGAG	GGGTCATCTTCTCTCTGTTGGC
OsNRT2.3b	GCCATCCACAAGATCGGTAG	TGTGGAGCTTCCCGTAGTTG
HvNRT2.1	TTGTGCACTTCCCACAATGG	GACCCTTGGCTTTCTCCTCT
HvNRT2.2	ACCCAGCTCATTATGCCACT	CCATCACCACGTGCATCATT
HvNRT2.3	GTGCCCTTTGGAACATCTGG	AGGGTGCGACACCAAAGATA
HvNAR2.3	AAGAAGGACAAGACCTGCCA	GTGATGCTGACGACGTTGAA

Table S3. The effect of 0.2 mM NH_4^+ treatment on the distribution of total N, P and Fe in shoots and roots.

0.2mM NH_4^+

Distribution ration of shoot (%)	WT	Ubi-1	Ubi-2	Ubi-3	RSs1-1	RSs1-2	RSs1-3
Total N	69.79a	72.48a	73.31a	78.63a	75.85a	71.30a	71.52a
Total P	56.07a	59.80a	62.80a	73.94a	70.27a	63.67a	60.61a
Total Fe	14.05b	9.73b	11.84b	26.25a	16.98b	17.11b	15.29b

Distribution ration of root (%)	WT	Ubi-1	Ubi-2	Ubi-3	RSs1-1	RSs1-2	RSs1-3
Total N	30.21a	27.52a	26.69a	21.37a	24.15a	28.70a	28.48a
Total P	43.93a	40.20a	37.20a	26.06a	29.73a	36.33a	39.39a
Total Fe	85.95a	90.27a	88.16a	73.75b	83.02a	82.89a	84.71a

Table S3. The effect of 0.2 mM NH_4^+ treatment on the distribution of total N, P and Fe in shoots and roots. Significant differences between transgenic and WT lines are indicated by different letters ($P < 0.05$, one-way ANOVA).

Table S4. Comparison of the expression of *OsNRT2.3b* in transgenic barley driven by different promoters under 0.2mM NH_4^+/NO_3^- treatment.

Leaves	Ubi-1	Ubi-2	Ubi-3	RSs1-1	RSs1-2	RSs1-3
0.2mM NH_4^+	0.000958e	0.001218e	0.001239e	0.000788e	0.000822e	0.000499e
0.2mM NO_3^-	0.018984c	0.035601b	0.079687a	0.009516d	0.009085d	0.013484d
Sheath	Ubi-1	Ubi-2	Ubi-3	RSs1-1	RSs1-2	RSs1-3
0.2mM NH_4^+	0.0000259d	0.000203d	0.000387d	0.000195d	0.000123d	0.000278d
0.2mM NO_3^-	0.0217b	0.0439a	0.0123c	0.0149c	0.0149c	0.0148c
Root	Ubi-1	Ubi-2	Ubi-3	RSs1-1	RSs1-2	RSs1-3
0.2mM NH_4^+	0.0000477c	0.000253c	0.000271c	0.000126c	0.000126c	0.0000588c
0.2mM NO_3^-	0.00404ab	0.004614a	0.001561bc	0.0000219c	0.0000851c	0.000447c

Table S4. Comparison of the expression of *OsNRT2.3b* in transgenic barley driven by different promoters under 0.2mM NH_4^+/NO_3^- treatment. Significant differences between NO_3^- and NH_4^+ condition in transgenic lines are indicated by different letters ($P < 0.05$, two-way ANOVA).

Figure S1. Identification of transgenic barley lines by southern blot.

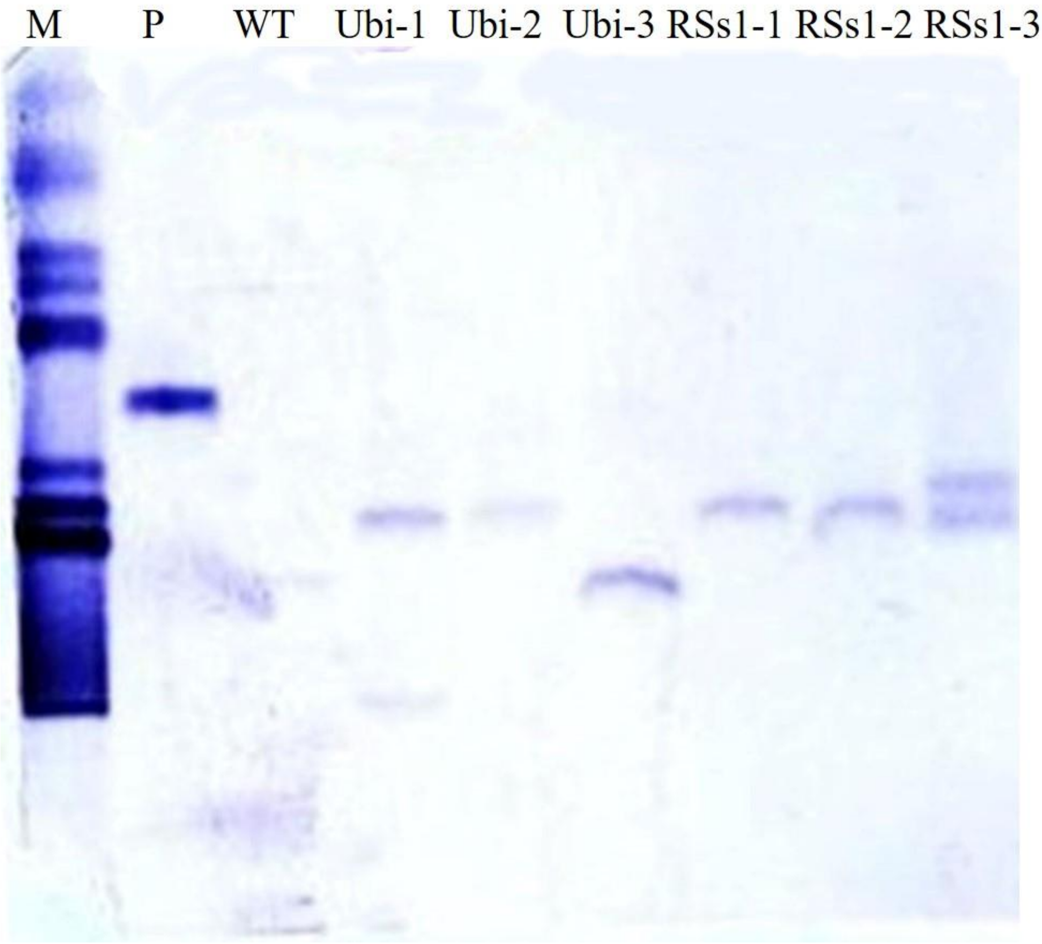

Fig. S1. Identification of transgenic barley lines by southern blot. Genomic DNA was isolated from WT and transgenic barley lines. Hybridization was performed using a hygromycin gene probe. M: maker, P: positive control.

Figure S2. The seed concentrations of Mn and Mg in different barley lines.

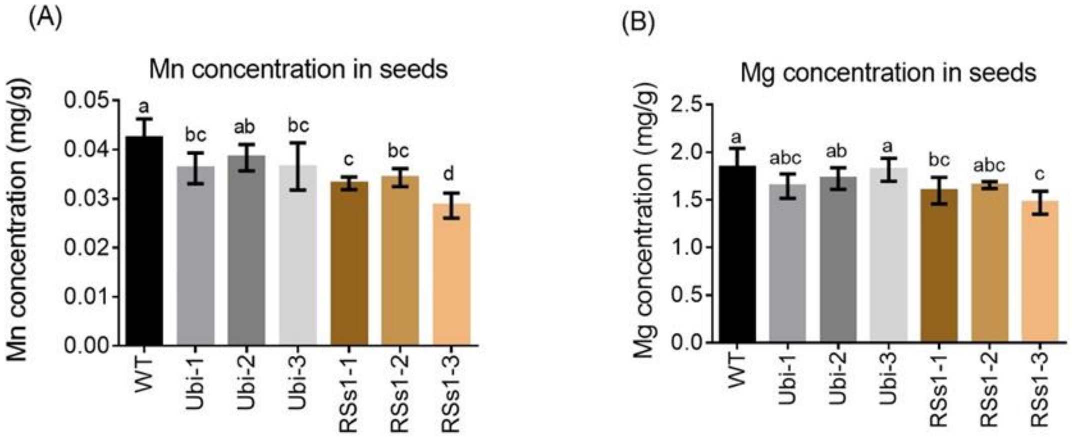

Fig. S2. The seed concentrations of Mn and Mg in different barley lines. (A) The concentration of Mn and (B) Mg in seeds. Significant differences between transgenic and WT lines are indicated by different letters ($P < 0.05$, one-way ANOVA). Error bars: standard error (n = 4 plants).

Figure S3. Homology analysis of *NRT2s* genes.

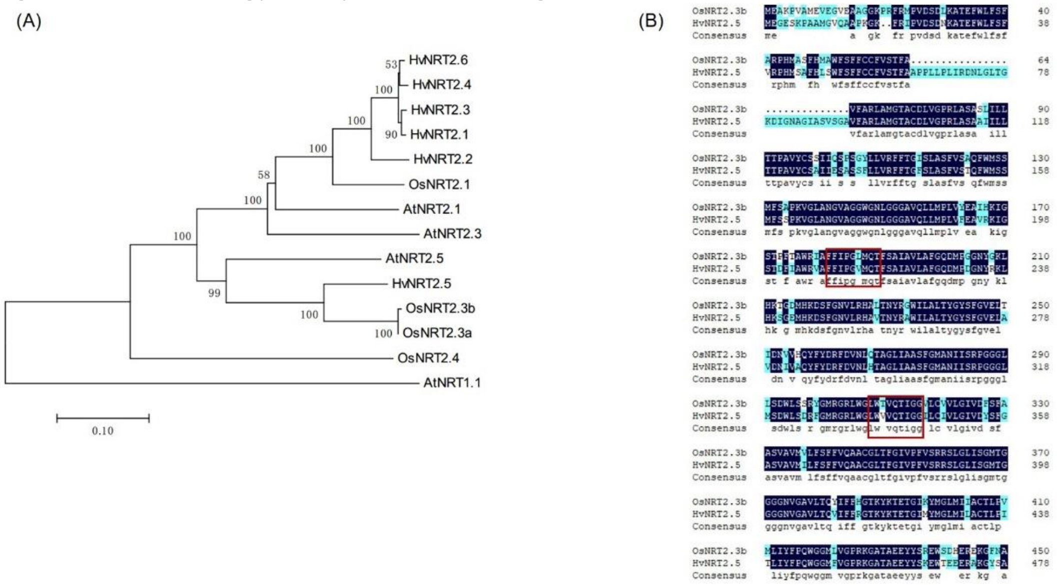

Fig. S3. Homology analysis of *NRT2s* genes. (A)The phylogenetic tree of the *NRT2* gene family in Arabidopsis, rice and barley. (B) HvNRT2.5 and OsNRT2.3b protein sequence alignment. The red box represented the pH sensing motif that was identified by OsNRT2.3b.

Figure S4. The relative expression of *HvNRT2s* in different plants parts under 0.2mM NH_4^+ and 0.2mM NO_3^- treatments.

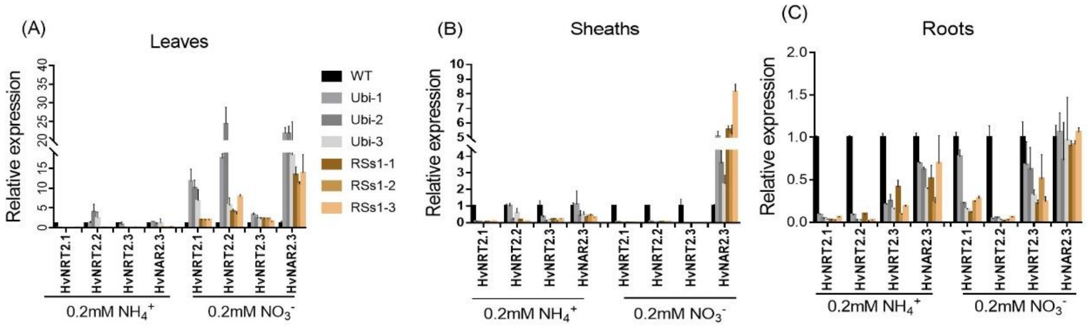

Fig. S4. The relative expression of *HvNRT2s* in different plants parts under 0.2mM NH_4^+ and 0.2mM NO_3^- treatments. The relative expression of *HvNRT2.1/2.2/2.3* and *HvNAR2.3* in (A) leaves, (B) sheaths and (C) roots of all barley lines under 0.2mM NH_4^+ and 0.2mM NO_3^- treatments. Significant differences between transgenic and WT lines are indicated by different letters ($P < 0.05$, one-way ANOVA). Error bars: standard error (n = 4 plants).

Figure S5. Phosphorus (P) and iron (Fe) concentrations in different plant parts under 0.2 mM NH_4^+ and 0.2mM NO_3^- treatments.

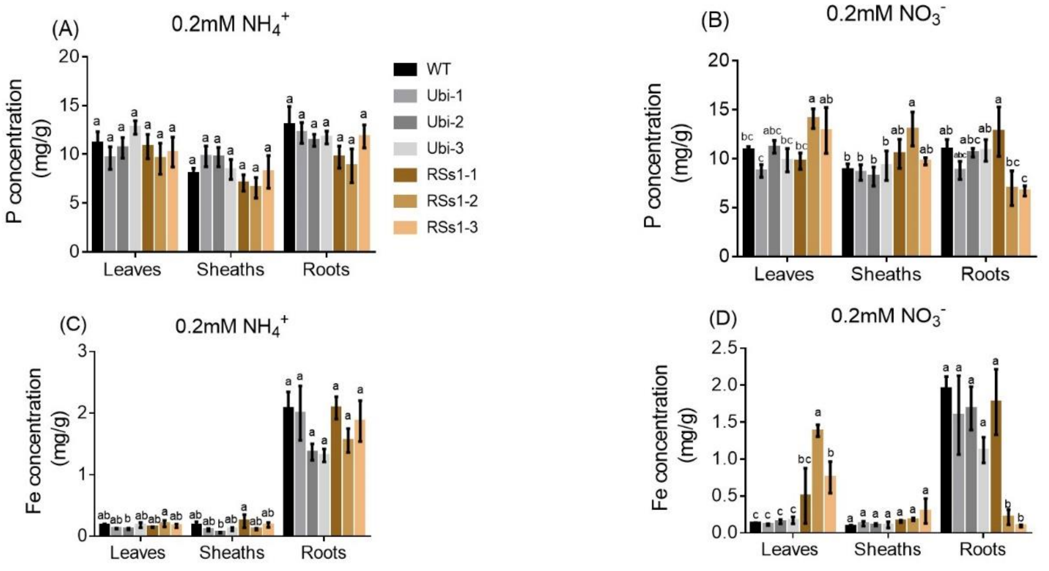

Fig. S5. Phosphorus (P) and iron (Fe) concentrations in different plant parts under 0.2 mM NH_4^+ and 0.2mM NO_3^- treatments. (A) The concentration of P and (C) Fe in leaves, sheaths and roots of all barley lines under 0.2 mM NH_4^+ condition. (B) The concentration of P and (D) Fe in leaves, sheaths and roots of all barley lines under 0.2mM NO_3^- condition. Significant differences between transgenic and WT lines are indicated by different letters ($P < 0.05$, one-way ANOVA). Error bars: standard error (n = 4 plants).

Figure S6. The characterization of all barley lines. Under 10 mM NH_4^+ condition after 10 mM NH_4^+/NO_3^- condition.

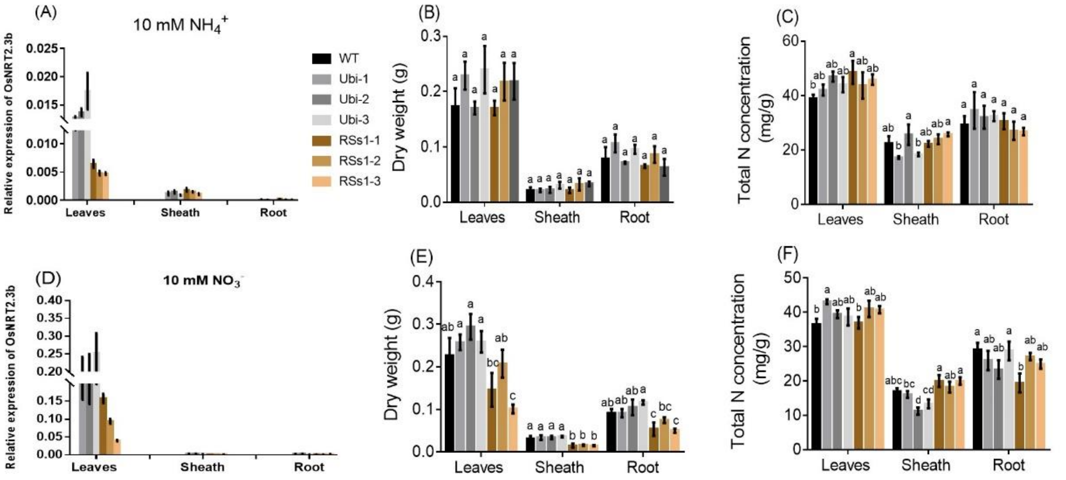

Fig. S6. The characterization of all barley lines. Under 10 mM NH_4^+ condition after 10 mM NH_4^+/NO_3^- condition. (A–C) 10 mM NH_4^+ (A) the relative expression of *OsNRT2.3b*, (B) the dry weights and (C) total N concentration of different plant parts. (D-F) 10 mM NO_3^-, (D) the relative expression of *OsNRT2.3b*, (E) the dry weight and (F) total N concentration of different plant parts. Significant differences between transgenic and WT lines are indicated by different letters ($P < 0.05$, one-way ANOVA). Error bars: standard error (n = 4 plants).
